# Transcriptional profile of the homologous recombination machinery and characterization of the EhRAD51 recombinase in response to DNA damage in *Entamoeba histolytica*

**DOI:** 10.1186/1471-2199-9-35

**Published:** 2008-04-10

**Authors:** Mavil López-Casamichana, Esther Orozco, Laurence A Marchat, César López-Camarillo

**Affiliations:** 1 Posgrado en Ciencias Genómicas, Universidad Autónoma de la Ciudad de México, México DF, México; 2 Departamento de Patología Experimental, CINVESTAV-IPN, México DF, México; 3 Programa Institucional de Biomedicina Molecular, ENMH-IPN, México DF, México

## Abstract

**Background:**

In eukaryotic and prokaryotic cells, homologous recombination is an accurate mechanism to generate genetic diversity, and it is also used to repair DNA double strand-breaks. *RAD52 *epistasis group genes involved in recombinational DNA repair, including *mre11, rad50, nsb1/xrs2, rad51, rad51c/rad57, rad51b/rad55, rad51d, xrcc2, xrcc3, rad52, rad54, rad54b/rdh54 *and *rad59 *genes, have been studied in human and yeast cells. Notably, the RAD51 recombinase catalyses strand transfer between a broken DNA and its undamaged homologous strand, to allow damaged region repair. In protozoan parasites, homologous recombination generating antigenic variation and genomic rearrangements is responsible for virulence variation and drug resistance. However, in *Entamoeba histolytica *the protozoan parasite responsible for human amoebiasis, DNA repair and homologous recombination mechanisms are still unknown.

**Results:**

In this paper, we initiated the study of the mechanism for DNA repair by homologous recombination in the primitive eukaryote *E. histolytica *using UV-C (150 J/m^2^) irradiated trophozoites. DNA double strand-breaks were evidenced in irradiated cells by TUNEL and comet assays and evaluation of the EhH2AX histone phosphorylation status. In *E. histolytica *genome, we identified genes homologous to yeast and human RAD52 epistasis group genes involved in DNA double strand-breaks repair by homologous recombination. Interestingly, the *E. histolytica *RAD52 epistasis group related genes were differentially expressed before and after UV-C treatment. Next, we focused on the characterization of the putative recombinase EhRAD51, which conserves the typical architecture of RECA/RAD51 proteins. Specific antibodies immunodetected EhRAD51 protein in both nuclear and cytoplasmic compartments. Moreover, after DNA damage, EhRAD51 was located as typical nuclear *foci*-like structures in *E. histolytica *trophozoites. Purified recombinant EhRAD51 exhibited DNA binding and pairing activities and exchanging reactions between homologous strands *in vitro*.

**Conclusion:**

*E. histolytica *genome contains most of the RAD52 epistasis group related genes, which were differentially expressed when DNA double strand-breaks were induced by UV-C irradiation. In response to DNA damage, EhRAD51 protein is overexpressed and relocalized in nuclear *foci*-like structures. Functional assays confirmed that EhRAD51 is a *bonafide *recombinase. These data provided the first insights about the potential roles of the *E. histolytica *RAD52 epistasis group genes and EhRAD51 protein function in DNA damage response of this ancient eukaryotic parasite.

## Background

*Entamoeba histolytica*, the protozoan causative of human amoebiasis, has a world-wide distribution with a higher prevalence in developing countries, affecting more than 50 million people each year [1]. Trophozoites show a dramatic virulence variability that could be related to great genome plasticity [2]. Frequent ploidy changes, unscheduled gene amplification and duplication have been reported [3,4], and it has been largely assumed that these processes are linked to genetic rearrangements, although no direct experimental evidence has been provided yet.

In eukaryotic and prokaryotic cells, homologous recombination (HR) is an accurate mechanism to generate genetic diversity. HR is also used by cells to properly repair the DNA double strand-breaks (DSBs). Generally, this kind of damage is produced by genotoxic agents or during cellular processes like meiotic division, telomere maintenance, and restoration of collapsed replication forks in the course of DNA synthesis [5-7]. Cellular response to DNA DSBs activates a complex network of proteins that transiently arrests cell cycle and enhances DNA repair mechanisms. Particularly, *Saccharomyces cerevisiae *H2A and *Homo sapiens *H2AX histones are rapidly phosphorylated in the chromatin micro-environment surrounding DNA DSBs, inducing nucleosome remodeling to promote accumulation of checkpoint and DNA repair proteins at these sites [8]. In case of extreme DNA damage, cells are targeted to apoptosis [9]. Additionally, HR is also a useful tool to analyze gene function by gene targeting and gene knock out approaches [10].

Molecular genetics of HR DNA repair has been well preserved throughout evolution. *RAD52 *epistasis group genes involved in DNA DSB repair, including *mre11, rad50, nsb1/xrs2, rad51, rad51c/rad57, rad51b/rad55, rad51d, xrcc2, xrcc3, rad52, rad54, rad54b/rdh54 *and *rad59 *genes, have been identified in human and yeast cells [11]. Pivotal protein in HR pathway is the RAD51 recombinase, which catalyses strand transfer between a broken DNA and its undamaged homologous strand, allowing damaged region to be repaired [12]. Strand exchange reaction is initiated by RAD51-coating of single-stranded DNA (ssDNA) released from DSBs, to generate a nucleoprotein filament. This active thread binds the intact double-stranded DNA (dsDNA) substrate, searching and locating homologous sequences, and promoting DNA strand exchange in an ATP-dependent manner, forming a heteroduplex structure called D-loop [13]. After DNA damage, RAD51 protein has been observed in nuclear complexes forming discrete *foci*, which are considered as the recombinational DNA repair sites [14].

HR remains the predominant mechanism to repair DSBs in lower eukaryotes [15]. RAD51 proteins have been identified in *Trypanosoma brucei *and *Plasmodium falciparum *parasites, which perform HR to switch the expression of genes encoding surface membrane glycoproteins and generate antigenic variation [16-18]. Furthermore, recombinational rearrangements are responsible for amplification of the multidrug resistance *pfmdr1 *gene in *P. falciparum *[19], demonstrating the relevance of HR to generate genomic versatility and plasticity in protozoan parasites.

In this paper, we identified and analyzed the mRNA expression profile of *E. histolytica *RAD52 epistasis group related genes in response to DNA damage. Additionally, we presented experimental evidence of EhRAD51 function as a recombinase, which suggest its potential role in DNA damage response. These findings constitute the initial efforts to understand the DNA repair mechanism in *E. histolytica *that will contribute to the further elucidation of events regulating genome integrity and variability in this early-branch protozoan.

## Results

### High dose of UV-C light induces DNA fragmentation in trophozoites

**Figure 1:**
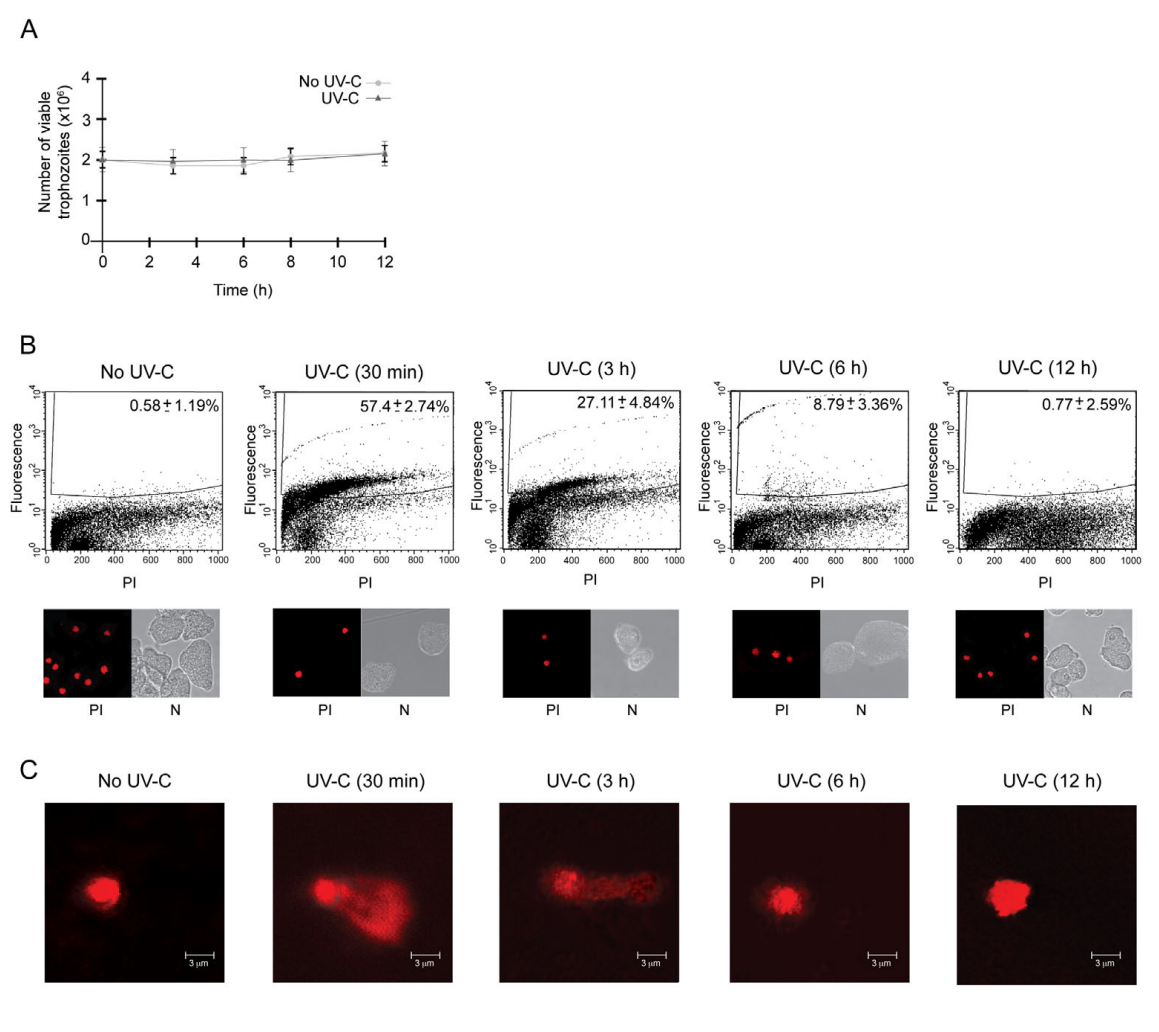
**Effect of UV-C irradiation on cell viability and DNA integrity of trophozoites**. **A**. Growth curves of non-irradiated and irradiated trophozoites (150 J/m^2 ^of UV-C light for 8 s). **B**. TUNEL assay and flow cytometry (FACS) assays of non-irradiated (No UV-C) and irradiated (UV-C) trophozoites harvested at different times (30 min, 3, 6 and 12 h). Upper panels, histograms show the DNA fragmentation percentage in fluorescence positive cells. The abscissa indicates fluorescence of propidium iodide (PI), and the ordinate indicates fluorescence of Alexa 488-labeled 3' ends of DNA. The number inside each histogram denotes the percentage of fluorescence positive cells above the cut-off line. Lower panels, PI-staining cells were checked in the epifluorescence microscope to confirming the absence of cytoplasmic stain. PI, propidum iodide, N, Nomanski optics. **C**. Neutral comet assays of non-irradiated (No UV-C) and irradiated (UV-C) trophozoites harvested at different times (30 min, 3, 6 and 12 h). Electrophoretic migration of DNA was from left (anode) to right (cathode).

It has been shown in a wide variety of cells that X-rays exposure, UV irradiation and chemicals activate cellular responses to DNA repair [20]. To initiate the study of the mechanisms involved in DNA repair in *E. histolytica*, we used UV-C light irradiation to induce DNA damage in trophozoites. Our experiments showed that during the first 12 h after irradiation with 254 nm UV-C (150 J/m^2^), cell survival was not significantly affected (Fig. 1A). Using the same experimental conditions, we analyzed the presence of 3'-hydroxyl DNA ends by TUNEL and FACS assays. In untreated trophozoites, FACS analysis evidenced the presence of <1% TUNEL positive cells; meanwhile, 30 min after treatment, 57.4 ± 2.74% of UV-C irradiated cells showed DNA fragmentation (Fig. 1B, upper panels). DNA damage reduction was observed at 3, 6 and 12 h after treatment (27.11 ± 4.84, 8.79 ± 3.36 and 0.77 ± 2.59%, respectively). Propidium iodide stained cells were checked under the fluorescence microscope to confirm the absence of cytoplasmic stain (Fig. 1B, lower panels).

The comet assay (single-cell gel electrophoresis) is widely used to measure DNA damage and repair. Results obtained through neutral comet assay (Fig. 1C) confirmed the induction of DSBs in trophozoites by UV-C treatment. Typical comet-like structures were observed at 30 min and 3 h, while a reduction of the DNA tails was observed at 6 h after UV-C treatment. As expected, 12 h after the genotoxic insult, DNA migration was similar to the control untreated cells (No UV-C). Taking altogether, these data indicated that UV-C irradiation efficiently induced DNA damage and consequently, repair mechanisms were activated to restore DNA integrity allowing cell survival.

### Early EhH2AX histone phosphorylation correlates with the presence of DNA DSBs

**Figure 2:**
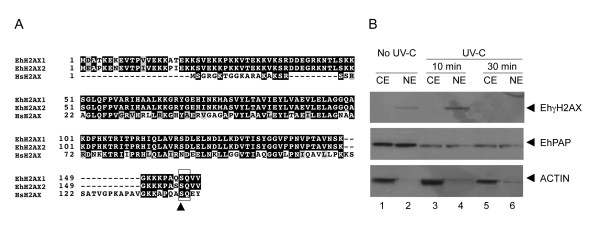
**Identification and immunodetection of phosphorylated EhH2AX histones (γEhH2AX)**. **A**. Multiple sequence alignments of *E. histolytica *and *H. sapiens *H2AX histones sequences. Black boxes, identical residues; grey boxes, conserved substitutions; open box, C-terminal SQ motif. Arrowhead denotes the potential phosphorylable serine residue (S156). Numbers at left are relative to the position of the initial methionine in each protein. **B**. Immunodetection of phosphorylated γEhH2AX polypeptides by Western blot assays using anti-human γH2AX polyclonal antibody (upper panel). CE, cytoplasmic extracts; NE nuclear extracts. Lanes 1 and 2, non-irradiated (No UV.C) trophozoites; lanes 3 and 4, irradiated trophozoites (10 min after UV-C treatment); lanes 5 and 6, irradiated (UV-C) trophozoites (30 min after UV-C treatment). Controls using anti-EhPAP and anti-actin antibodies (middle and bottom panels, respectively) are shown.

DNA DSBs induce early phosphorylation of yeast H2A (major H2A closer to mammalian H2AX) and human H2AX histones on a conserved serine residue located in the SQ motif at C terminus, producing γH2A and γH2AX, respectively [21]. As in yeast, *E. histolytica *seems to have replaced the canonical H2A with H2AX [22]. Two genes (locus EHI_126210 and EHI_188960) that encode putative proteins with 55 and 57% identity (*e-*value 2e-27and 2e-28) to yeast H2A and human H2AX histones, respectively, were found in the *E. histolytica *genome. These genes predict two 17.6 kDa conserved paralogous H2AX proteins that share 93% identity. Notably, both contain the H2AX exclusive SQ motif with the potentially phosphorylable serine residue (S156) (Fig. 2A).

Taking advantage of the high conservation between *H. sapiens *and *E. histolytica *H2AX C-terminus, we performed Western blot assays using the anti-human γH2AX antibody to detect serine-phosphorylated EhH2AX homologues (γEhH2AX) in cytoplasmic (CE) and nuclear (NE) extracts of trophozoites. Protein amount and integrity were confirmed on Coomassie blue stained-gels (data not shown). In NE from non-irradiated cells, we identified a 17-kDa weak band, which corresponds to the expected molecular weight of γEhH2AX histones (Fig. 2B, lane 2). Interestingly, 10 min after UV-C irradiation, this band was five-fold more intense, suggesting an increase in the amount of nuclear γEhH2AX, and 30 min after treatment no band was found (Fig. 2B, lanes 4 and 6). However, these assays did not allow us to distinguish whether one or both EhH2AX proteins were phosphorylated. In contrast, no signals were observed in CE (Fig. 2B, lanes 1, 3 and 5). We used as an integrity control an anti-EhPAP serum, which recognized the 63-kDa EhPAP protein [23] in non-irradiated and irradiated trophozoites (Fig. 2B, middle panel). In addition, an anti-actin monoclonal antibody, used as control for cell fractionation, strongly detected the expected 42-kDa band in CE and a slight signal in NE, as expected for a major component of cytoskeleton (Fig. 2B, lower panel). These data showed that UV-C irradiation of trophozoites is a useful model to generate DNA DSBs and study DNA repair in *E. histolytica*.

### *E. histolytica *genome contains RAD52 epistasis group related genes

In order to investigate the presence of RAD52 epistasis group related genes in *E. histolytica *genome, we surveyed the parasite Pathema database (Table 1). We found *Ehmre11, Ehrad50 *and *Ehnbs1 *genes, which could encode the *E. histolytica *putative MRE11-RAD50-NBS1 protein complex that functions as the primary sensor of DNA DSBs in other organisms [9]. Both EhMRE11 and EhRAD50 proteins exhibited 32 to 23% identities (*e*-values from 3e-49 to 9e-36) with *S. cerevisiae *and *H. sapiens *orthologous proteins, respectively; whereas the EhNBS1 sequence appears to be more divergent (17 to 24% identity and *e*-values from 0.003 to 0.002). *E. histolytica *also contains genes encoding the putative recombinase EhRAD51 and its paralogous protein EhRAD51C. EhRAD52, EhRAD54, EhRAD54B and EhRAD59 (EhRAD52/22 in Pathema database) predicted proteins are also encoded in the *E. histolytica *genome. As in yeast, *rad51 *paralogs (*rad51b*, *rad51d, xrcc2 *and *xrcc3*) that participate in HR in vertebrates were not found in *E. histolytica *(Table 1). In conclusion, *E. histolytica *genome contains a conserved set of repair genes, which suggests that it is skilled to perform recombinational DNA repair.

**Table 1:** Comparison of *E. histolytica*, *H. sapiens *and *S. cerevisiae *RAD52 epistasis group proteins

*Entamoeba histolytica*				*Homo sapiens*						*Saccharomyces cerevisiae*					
Predicted protein	Size (aa)	GeneBank ID	Locus name ^a^	Protein	Accession number ^b^	Size (aa)	e-value	H (%)	I (%)	Protein	Accession number ^b^	Size (aa)	e-value	H (%)	I (%)

EhMRE11	596	XM_651393	EHI_125910	MRE11	P49959	708	3e-49	51	32	MRE11	P32829	692	1e-33	45	26
EhMRE11-like	223	XM_644963	EHI_077650	MRE11	P49959	708	3e-23	51	25	MRE11	P32829	692	2e-20	49	27
EhRAD50	1241	XM_647783	EHI_079960	RAD50	Q92878	1312	2e-44	40	23	RAD50	P12753	1312	2e-43	46	27
EhNBS1	764	XM_647447	EHI_098770	NBS1	Q6IQ31	754	0.0022	40	24	XRS2	P33301	854	e+3	28	17
EhRAD51	367	XM_648984	EHI_031220	RAD51	Q06609	339	e-125	83	71	RAD51	P25454	400	2e-43	78	60
EhRAD51C	284	XM_619126	EHI_122860	RAD51C	Q433502	376	3e-17	51	28	RAD57	P25301	460	e-109	47	27
-	-	-		RAD51B	O15315	350	-	-	-	RAD55	P38953	406	-	-	-
-	-	-		RAD51D	O75771	289	-	-	-	-	-	-	-	-	-
-	-	-		XRCC2	O43543	280	-	-	-	-	-	-	-	-	-
-	-	-		XRCC3	O43542	346	-	-	-	-	-	-		-	-
EhRAD52	243	XM_648599	EHI_188230	RAD52	P43351	418	2e-35	71	48	RAD52	P06778	504	1e-23	57	38
EhRAD54	885	XM_648260	EHI_103840	RAD54	Q92698	747	e-115	58	41	RAD54	P32863	898	e-106	56	37
EhRAD54B	765	XM_645236	EHI_114930	RAD54B	Q9Y620	910	e-130	52	36	RDH54	P32863	920	e-114	55	39
EhRAD59 (EhRAD52/22^a^)	190	XM_651011	EHI_112840	-	-	-	-	-	-	RAD59	Q12223	238	2e-08	45	25

^a ^*E. histolytica *Pathema datatabase

^b ^Swiss-Prot/TrEMBL databases

I, identity; H, homology

### *E. histolytica *genes of the RAD52 epistasis group are differentially expressed in response to UV-C irradiation

**Figure 3:**
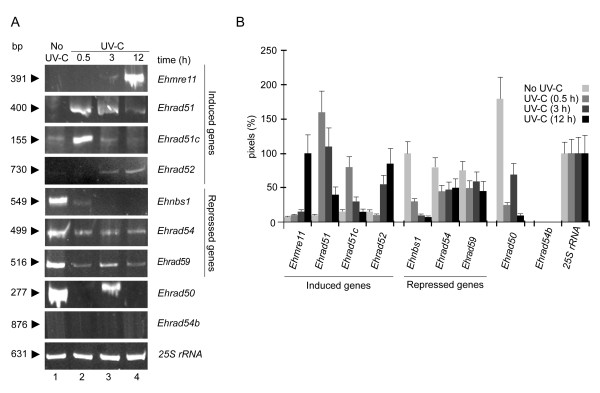
**mRNA expression profiles of *E. histolytica *RAD52 epistasis group related genes**. **A**. Ethidium bromide stained 6% PAGE showing the RT-PCR products obtained from 1 μg of total RNA of non-irradiated (No UV-C, lane 1) or irradiated trophozoites harvested at different times (UV-C; lane 2, 0.5 h; lane 3, 3 h and lane 4,12 h). Arrowheads denote the length (bp) of each expected amplified internal fragment, as described in Table 2. **B**. Densitometric analyses of RT-PCR products in A. Pixels corresponding to the *25S *rRNA product were taken as 100% in each lane. Data are the mean of three independent assays.

As a first step towards establishing the role of the *E. histolytica *RAD52 epistasis group related genes, we evaluated their mRNA expression by semi-quantitative RT-PCR using the UV-C irradiation model described above. Most genes exhibited a differential mRNA expression profile before and after irradiation (Fig. 3). *Ehmre11, Ehrad51, Ehrad51c *and *Ehrad52 *genes were transcribed at a very low level in non-irradiated trophozoites; meanwhile mRNA expression was induced from 30 min to 12 h after genotoxic damage. Particularly, the *Ehrad51 *mRNA expression was 16-, 11- and 4-fold increased at 30 min, 3 h and 12 h, respectively, after UV-C irradiation, when compared with untreated cells (Fig. 3A and 3B). On the other hand, the *Ehnbs1*, *Ehrad54 *and *Ehrad59 *genes were abundantly transcribed in untreated trophozoites; however, mRNA levels were down-regulated after UV-C treatment. The *Ehrad50 *gene expression showed the highest steady-state mRNA levels in non-irradiated trophozoites. At 30 min after UV-C irradiation, *Ehrad50 *transcript levels dropped drastically; 3 h later, they moderately increased, and at 12 h they diminished again. In contrast, *Ehrad54b *gene did not seem to be expressed under the experimental conditions tested here (Fig. 3A and 3B). We observed minimal changes in the *25S rRNA *expression used as control (Fig. 3A, lower panel). These data showed that *E. histolytica *RAD52 epistasis group related genes were differentially expressed in response to DNA damage.

### The predicted EhRAD51 conserves the typical architecture of RECA/RAD51 family members

**Figure 4:**
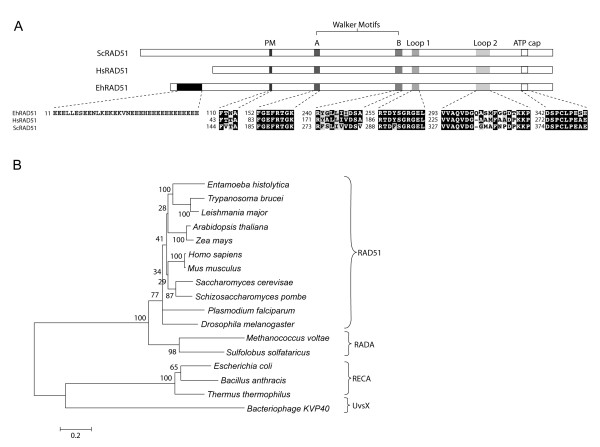
**Comparison of the predicted amino acids sequence of EhRAD51 with other RAD51 proteins**. **A**. Multiple sequence alignments of functional domains of RAD51 from *E. histolytica *(EhRAD51), *S. cerevisiae *(ScRAD51) and *H. sapiens *(HsRAD51) proteins. Upper panel: Glutamate-rich region, polymerization motif (PM), Walker A and B motif, L1 and L2 regions and ATP cap appear as colored boxes. Lower panel: black boxes, identical aa; grey boxes, conserved substitutions; open box, Glutamate-rich region. Numbers at the left are relative to the position of the initial methionine in each protein. Accession numbers and protein lengths are indicated in the Supplementary Table S1. **B**. Phylogenetic relationships between EhRAD51 and RECA/RAD51 family members. The unrooted tree was created with the MEGA 3.1 program using the Neighbor Joining algorithm based on ClustalW alignments of complete amino acids sequences. Numbers above the tree nodes indicate the percentage of times that the branch was recovered in 1000 replications.

Since RAD51 recombinases are considered as key enzymes in HR and DNA repair processes in many organisms [24], we focused on the characterization of the *E. histolytica *EhRAD51 protein. *Ehrad51 *is an intron-less 1101 bp gene, which encodes a 367 amino acids (aa) polypeptide (40.3-kDa). Sequence similarity searches by BLAST showed the lowest *e-*values (from 3e-29 to 2e-20) and high identity (from 59 to 75%) with many eukaryotic RAD51 proteins, from plants to human, including protozoan parasites. Moreover, EhRAD51 showed 51% and 36% identity with *Methanococcus voltae *RADA and *Escherichia coli *RECA bacterial recombinases, respectively (Additional file 1). Amino acid sequence alignment of EhRAD51 protein with yeast and human RAD51 orthologs revealed that these proteins share functional and structural conserved motifs (Fig. 4A). EhRAD51 contains the putative polymerization motif (110–113 aa residues), which tethers individual subunits to form quaternary assemblies in human RAD51 protein [24] (Additional file 2). We also identified the ATPase Walker A or phosphate binding loop (P-loop: 152–159 aa residues) and Walker B motifs (240–249 aa residues), the ssDNA binding loops L1 (255–264 aa residues) and L2 (293–311 aa residues), as well as the ATP-stacking motif or ATP cap (342–350 aa residues) at the C-terminus, which are essential for nucleofilament assembling and ATP hydrolysis in RAD51/RECA-like recombinases [26,27]. Remarkably, the EhRAD51 N-terminus has a low-complexity region of 34-aa highly enriched in glutamic residues, which is not present in homologous proteins (Fig. 4A). Phylogenetic relationships among EhRAD51 and RAD51/RECA related proteins from diverse organisms, revealed a progressive evolution from eubacteria to eukaryotes, being EhRAD51 more related to protozoan recombinases (Fig. 4B).

### The EhRAD51 protein is overexpressed in response to DNA damage

**Figure 5:**
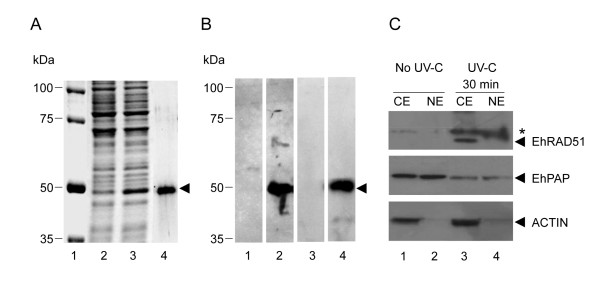
**Expression and immunodetection of EhRAD51**. **A**. Expression and purification of rEhRAD51-6x His-tagged protein. Bacterial proteins were separated through 10% SDS-PAGE and gels were stained with Coomassie blue. Lane 1, molecular weight markers; lane 2, non-induced bacterial extract (30 μg); lane 3, IPTG-induced bacterial extract (30 μg) before passing through the Ni^2+^-NTA affinity column; lane 4, affinity purified polypeptide from IPTG-induced bacteria extract. Arrowhead, 47-kDa rEhRAD51. **B**. Immunodetection of rEhRAD51 polypeptide. Western blot assays were performed using non-induced bacterial extract (lane 1) and purified rEhRAD51 (lanes 2 to 4). Lanes 1 and 2; anti-6x His tag antibodies; lane 3, preimmune serum; lane 4, specific rabbit antibodies raised against rEhRAD51. Arrowhead, 47-kDa rEhRAD51. **C**. Immunodetection of *E. histolytica *endogenous EhRAD51 by Western blot assays using specific anti-EhRAD51 antibodies. CE, cytoplasmic extracts; NE nuclear extracts. Lanes 1 and 2, non-irradiated (No UV-C) trophozoites; lanes 3 and 4, irradiated (UV-C) trophozoites (30 min after UV-C treatment). Upper panel: arrowhead, 41-kDa EhRAD51; asterisk, 46-kDa EhRAD51.Controls using anti-EhPAP and anti-actin antibodies (middle and bottom panels, respectively) are shown.

The recombinant EhRAD51 protein (rEhRAD51) was expressed in *E. coli *BL21 (DE3) plysS strain as a 6x His-tagged fusion polypeptide and subsequently purified by affinity chromatography (Fig. 5A, lanes 3 and 4). By Western blot assays using monoclonal anti-6xHis tag antibodies, the purified rEhRAD51 was detected as a single 47 kDa band, which was slightly higher than the 44.1 kDa expected weight (Fig. 5B, lane 2). Then, rEhRAD51 was used to generate rabbit polyclonal anti-EhRAD51 antibodies. These antibodies recognized the 47 kDa rEhRAD51 band (Fig. 5B, lane 4), whereas the preimmune serum, used as negative control, did not detect any signal (Fig. 5B, lane 3). To evaluate the expression of the native EhRAD51 in *E. histolytica*, we performed Western blot assays using anti-EhRAD51 antibodies and protein extracts from irradiated and non-irradiated trophozoites. Antibodies reacted with a weak 46 kDa band in CE from non treated trophozoites, but not signal was detected in NE (Fig. 5C, higher panel, lanes 1 and 2). Meanwhile, at 30 min after UV-C irradiation, antibodies strongly detected the expected 41 kDa endogenous EhRAD51 in CE, but not in NE (Fig. 5C, higher panel lanes 3 and 4). Intriguingly, antibodies also detected a 46 kDa band in both NE and CE from UV-C irradiated trophozoites, which may correspond to a modified form of the 41 kDa protein. The specificity of anti-EhRAD51 antibodies was confirmed performing a similar Western blot assay using anti-EhRAD51 antibodies previously pre-incubated with purified rEhRAD51 protein and the recognition of both 46 and 41 kDa proteins was specifically inhibited (data not shown). In addition, the use of anti-EhPAP and anti-actin antibodies confirmed protein integrity and cell fractionation of CE and NE (Fig. 5C, middle and lower panels). Our findings showed that EhRAD51 was overexpressed in response to UV-C irradiation, and distributed in both nuclear and cytoplasmic compartments.

### EhRAD51 relocalizes into nuclear foci-like structures in response to DNA damage

**Figure 6:**
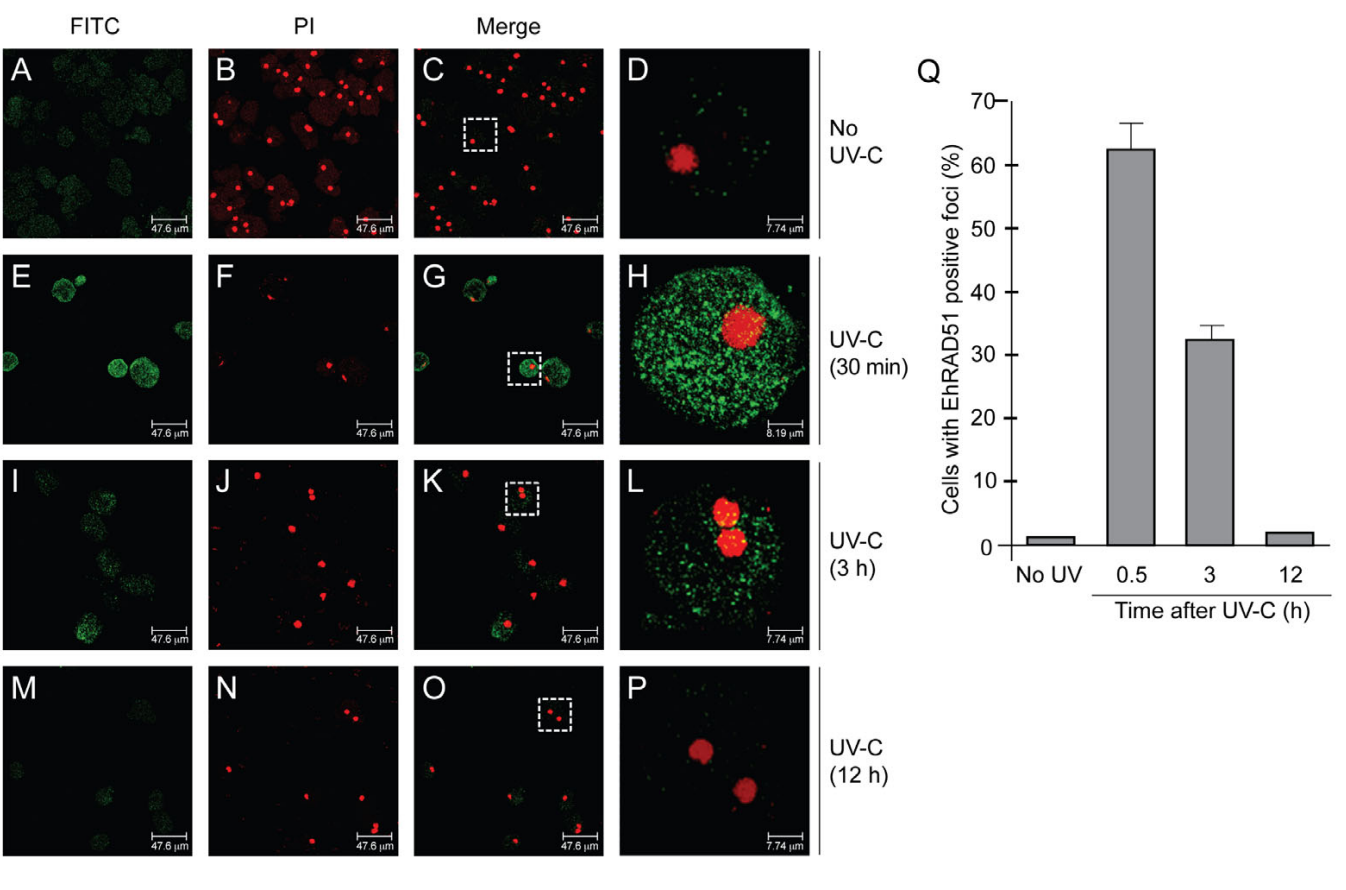
**Cellular localization of EhRAD51 in non-irradiated (No UV-C) and irradiated (UV-C) trophozoites at 30 min, 3 h and 12 h after treatment**. Trophozoites were incubated with anti-rEhRAD51 antibodies, treated with FITC-labeled secondary antibodies, counterstained with propidium iodide (PI) and analyzed through confocal immunofluorescence microscopy. **A-P. **EhRAD51 immunodetection. A, E, I and M, green channel (FITC); B, F, J and N, red channel (PI) channel; C, G, K and O, merge images; D, H, L and P, single cell (100× magnification) from boxes denoted in C, G, K and O.**Q**. Occurrence of EhRAD51 nuclear *foci*. The percentage of trophozoites displaying EhRAD51 *foci *was calculated after scoring 50 nuclei for each time point. Data are the mean of three independent assays.

In order to better characterize the EhRAD51 expression and function, we investigated its subcellular location in trophozoites through immunofluorescence and laser confocal microscopy. In agreement with the Western blot results, EhRAD51 was detected at low levels in the cytosol of non-irradiated trophozoites (Fig. 6, panels A-D), whereas at 30 min after UV-C irradiation we noted a dramatic accumulation of cytoplasmic EhRAD51 protein. Interestingly, we also observed a scattered distribution of EhRAD51 typical *foci*-like structures in the nucleus (Fig. 6, panels E-H). Three hours later, the cytoplasmic signal diminished, while nuclear *foci*-like structures remained (Fig. 6, panels I-L). At 12 h after genotoxic damage, both cytoplasmic and nuclear EhRAD51 signals were very weak, being EhRAD51 *foci*-like structures scarce (Fig. 6, panels M-P). Quantification of nuclear *foci *like-structures by statistical microscopic analysis showed that about 60% of the cells contained at least one *focus *at 30 min after UV-C irradiation (Fig. 6Q). These findings confirmed that EhRAD51 was up-regulated after UV-C irradiation and suggested that it was redistributed into the nucleus during the first 3 h after DNA damage.

### rEhRAD51 exhibits DNA binding activity *in vitro*

**Figure 7:**
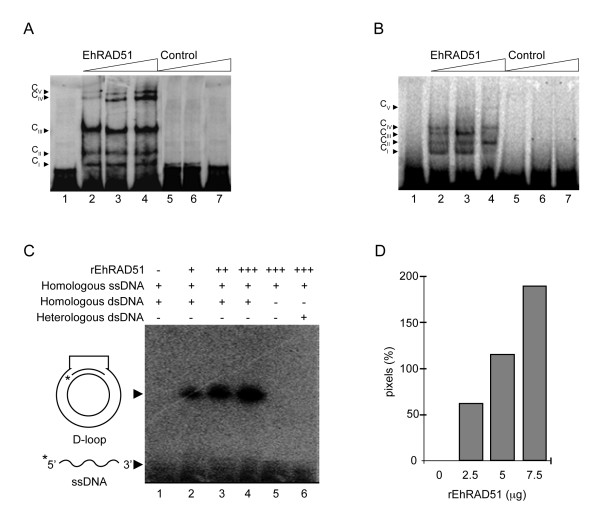
**DNA-binding and homologous strand transfer activities of rEhRAD51**. **A**. Partially-purified rEhRAD51 was incubated with [γ-^32^P]dATP labeled ssDNA and interactions were resolved through non-denaturing PAGE. Lane 1, free probe. Lanes 2 to 4, ssDNA incubated with increasing amounts of rEhRAD51 (2.5, 5 and 7.5 μg, respectively); lanes 5 to 7, ssDNA incubated with increasing concentrations of mock purified fraction (2.5, 5 and 7.5 μg) as control. Protein-DNA complexes (C_I _to C_V_) are shown by arrowheads. **B**. Partially purified rEhRAD51 was incubated with [α-^32^P]dATP labeled dsDNA and interactions were resolved through PAGE. Lane 1, free probe. Lanes 2 to 4, dsDNA incubated with increasing amounts of rEhRAD51 (2.5, 5 and 7.5 μg, respectively); lanes 5 to 7, dsDNA incubated with increasing concentrations of mock purified fraction *E. coli *elution fraction (2.5, 5 and 7.5 μg) as control. Protein-DNA complexes (C_I _to C_V_) are shown by arrowheads. **C**. D-loop reactions containing 10,000 cpm of [γ-^32^P]dATP-labeled oligonucleotide, circular dsDNA and 0, 2.5, 5 and 7.5 μg of partially-purified rEhRAD51 (lanes 1 to 4) were incubated at 37°C for 30 min with 2 mM of ATP. Negative controls were performed without homologous dsDNA (lane 5) and with heterologous dsDNA oligonucleotide instead of homologous dsDNA (lane 6), both of them using 7.5 μg of EhRAD51 elution fraction. Reaction products were analyzed by agarose gel electrophoresis, transferred to nylon membranes and visualized through a Phosphor Imager. **D**. Densitometric analysis of D-loop products obtained in C. Results are representative of two independent experiments.

*In silico *analysis of the EhRAD51 aa sequence evidenced the presence of two putative DNA binding domains. To verify that EhRAD51 is a DNA binding protein, we performed EMSA using increasing amounts of purified rEhRAD51 protein and a fixed concentration of radiolabeled 50-bp ssDNA or 270-bp dsDNA fragments as probes. In order to discard interactions of contaminant *E. coli *proteins with DNA probes, we used mock purified fractions obtained from untransformed bacteria as a negative control. Results showed that incubation of rEhRAD51 with ssDNA and dsDNA probes resulted in five DNA-protein complexes (C_I_-C_V_) formation, suggesting that alternative populations of RAD51 protomers were associated to each DNA probes (Fig. 7A and 7B, lanes 2 to 4). The fastest migration ssDNA-protein complex C_I _that was also formed with the mock fraction was considered as unspecific (Fig. 7A, lanes 5 to 7). No complexes were formed in the EMSA control performed with the dsDNA probe (Fig. 7B, lanes 5 to 7). Notably, the abundance of slow migration DNA-protein complexes appeared to increase in the presence of the highest rEhRAD51 amount (Fig. 7A and 7B, lanes 2 to 4). These results showed that rEhRAD51 was able to efficiently bind both ssDNA and dsDNA substrates *in vitro*.

### rEhRAD51 exhibits homologous DNA strand transfer activity *in vitro*

In order to evaluate the homologous DNA strand transfer function of the rEhRAD51 protein, we performed a pairing assay involving the D-loop structure formation as described in Experimental procedures. Results revealed that rEhRAD51 was able to shift the electrophoretic mobility of the radioactive-labeled 200-bp ssDNA probe incubated with homologous circular dsDNA (Fig. 7C, lanes 2 to 4). This indicated that rEhRAD51 was able to catalyze ssDNA transfer to homologous dsDNA forming the three-stranded pairing molecule or D-loop structure, which has a reduced electrophoretic mobility in comparison with the ssDNA probe. The D-loop formation specificity was confirmed by incubation of rEhRAD51 and ssDNA probe in the absence of homologous dsDNA substrate (Fig. 7C, lane 5), and in the presence of a heterologous dsDNA substrate (Fig. 7C, lane 6), since no complex was observed. In addition, we did not observe any D-loop structure in the absence of rEhRAD51 (Fig. 7C, lane 1). Densitometric analysis of radioactive products showed that D-loop structure formation using 7.5 μg of rEhRAD51 was 3.6 and 1.7-fold higher than with 2.5 and 5 μg of rEhRAD51, respectively (Fig. 7D). These results indicated that EhRAD51 protein was able to catalyze specific DNA paring and exchange between DNA homologous strands *in vitro*.

## Discussion

While non-homologous end joining plays a major role in DSB DNA repair in higher eukaryotes including mammals, HR remains the predominant mechanism to repair this kind of lesions in lower eukaryotes [15]. The high amount of repetitive DNA in protozoan parasites, such as *E. histolytica*, suggests that the genome of these organisms can be potentially recombinogenic. Therefore, the study of HR process in *E. histolytica *may advance our understanding about trophozoites genetic and virulence variability, as well as DNA repair mechanisms.

Here, we developed a 254 nm UV-C light irradiation model, which induces DNA damage in *E. histolytica *trophozoites and activates recombinational DNA repair pathway. Irradiation dose (150 J/m^2^) and time (8 s) were determined as no lethal conditions for cells in comparison with other UV doses previously evaluated. Growth curves were performed up to 18 h, the doubling time of trophozoites, without observing any significant changes (data not shown). Early phosphorylation of *E. histolytica *H2AX histones after UV-C irradiation was consistent with DNA DSBs formation, suggesting chromatin remodeling and recruitment of histone-phosphorylating enzymes, as observed in other eukaryotic systems [22]. Moreover, *E histolytica *trophozoites survival throughout almost 12 h after irradiation indicated the existence and activation of DNA repair mechanisms. *In silico *analysis of the *E. histolytica *genome sequence revealed that this pathogen has genes that encode putative EhRAD52 epistasis group members, which participate in recombinational DNA repair in other organims. Given the place of this ancient protista in the eukaryote phylogenetic scale, EhRAD52 epistasis group had equivalent similarity with homologous proteins from different organisms, such as mammals, plants and other protozoan parasites.

RT-PCR assays evidenced a differential mRNA expression of *E. histolytica rad52 *epistasis group genes, before and after DNA damage. Some genes (*Ehnbs1*, *Ehrad54 *and *Ehrad52/22*) were down-regulated after DNA damage, others (*Ehmre11*, *Ehrad51, Ehrad51-C and Ehrad52*) were up-regulated at different times following genotoxic stimulus, whereas *Ehrad50 *mRNA levels were regulated in a variable manner, suggesting a complex transcriptional response. Interestingly, *Ehrad54b *gene did not seem to be transcribed under our experimental conditions. However, in yeast and human, both RAD54 and RAD54B are DNA helicases which participate in the formation of heteroduplex DNA in recombination processes [11]. It is possible that the expression of *Ehrad54 *homolog is sufficient to cover this activity in trophozoites, although additional experiments are required to confirm this hypothesis. The absence of a coordinated transcriptional activation of *Ehrad52 *epistasis group genes suggest that trophozoites have enough stationary levels of enzymes for DBB repair and the main regulation could be occurring at translational and/or posttranslational level. A further evaluation of EhRAD52 epistasis group proteins regulation in response to DNA damage will help us to better understand DNA repair by HR in *E. histolytica*. It seems that the molecular events related to DNA lesions produced by genotoxic agents can be barely inferred from gene expression profiling. Indeed, studies in yeast and mammals have shown no-relationship between genes whose expression is increased after different DNA-damaging treatments (ionizing radiation, UV light, cisplatin, H_2_O_2_) and those genes that are involved in protecting against cytotoxicity to the same agents [28,29].

We focused on *Ehrad51 *gene because RAD51 proteins have been demonstrated as key players in recombinational DNA repair in lower and higher eukaryotes [for review see [12]]. Interestingly, the *Ehrad51 *transcript steady state levels were about 15-fold higher at 30 min post-UV-C treatment and decreased 3 and 12 h later, suggesting that EhRAD51 could be participating in HR in the early steps of DNA repair. Similar transcriptional activation after UV treatment has been reported as a common characteristic for *recA/rad51 *homologs of *Tetrahymena thermophila *[30] and *Halobacterium sp*. [31]. In agreement with the RT-PCR results, Western blot assays showed a dramatic increase of EhRAD51 in cytoplasm and nucleus, 30 min after DNA breaks were introduced into the *E. histolytica *genome. The fact that specific polyclonal antibodies immunodetected a 46 kDa EhRAD51 protein suggest that some posttranslational modifications of the cytoplasmic 41 kDa EhRAD51 could be a requirement for its translocation to the nucleus where DNA repair takes place. Taking in consideration that the EhRAD51 sequence lacks a nuclear localization signal, an alternative possibility might be that EhRAD51 needs to interact with other protein(s) to be transported inside the nucleus. However, additional experiments are required to corroborate these hypotheses.

As observed for yeast and human homologs [32], laser confocal microscopy evidenced focal sites of the EhRAD51 protein scattered in the nucleus at 30 min and 3 h after DNA damage. Congruently, the EhRAD51 nuclear *foci*-like structure occurrence was consistent with the DNA fragmentation degree observed in TUNEL and neutral comet assays. Since UV-C treatment did not affect trophozoites viability, it is tempting to suggest that DNA repair mechanisms involving EhRAD51 *foci *formation were activated to restore genome integrity after genotoxic insult.

*In silico *analysis demonstrated that the predicted EhRAD51 protein contains all functional and structural motifs that are important for RECA/RAD51 recombinases activities. To experimentally support its role in DNA repair by HR, we performed the basic characterization of EhRAD51 protein. EhRAD51 functional properties were similar to those previously reported for RAD51 homologous [33-35]. EhRAD51 was able to bind both ssDNA and dsDNA substrates in the presence of ATP and Mg^2+^. The various rEhRAD51-DNA complexes may be related to different amounts of rEhRAD51 molecules bound to ssDNA or dsDNA probe. Finally, EhRAD51 promoted specific three-stranded pairing structure formation or D-loop. Based on the data presented here, we proposed a working model for DNA DSB repair involving the EhRAD51 recombinase. When a DSB is introduced in *E. histolytica *genome, EhH2AX histones become phosphorylated, which could induce chromatin remodeling and accumulation of the EhRAD52 epistasis group proteins at the DNA DSB site. We observed that EhRAD51 was relocated into the DNA repair nuclear *foci*, where it could mediate DNA paring and homologous strand exchange to restore genome integrity. It is also possible that *E. histolytica *RAD51 protein may play a role in genome rearrangements that naturally occur within this organism during DNA synthesis. Therefore, it will be interesting to evaluate its involvement in frequent ploidy changes, unscheduled gene amplification and duplication events observed in *E. histolytica *genome [3,4]. Our next challenge will involve studying *in vivo *HR and the relevant role of EhRAD51 in this process in *E. histolytica*.

## Conclusion

Our results provide the first data supporting the role of the RAD52 epistasis group genes in DNA repair process in *E. histolytica*. We showed that *E. histolytica *RAD52 epistasis group genes, were differentially expressed when DNA fragmentation was induced by UV-C irradiation. We also showed that EhRAD51 protein was overexpressed and relocalized in nuclear *foci*-like structures after DNA damage, and demonstrated that recombinant EhRAD51 function as a recombinase *in vitro*. These data evidenced a potential role of EhRAD51 protein in DNA damage response in this ancient eukaryotic parasite.

## Methods

### *E. histolytica *cultures

Trophozoites of *E. histolytica *clone A (strain HM1: IMSS) were axenically cultured in TYI-S-33 medium [36] at 37°C and harvested during exponential growth phase.

### Trophozoites UV-C light irradiation

Trophozoites (2 × 10^6^) grown in culture bottles were transferred into glass dishes and incubated at 37°C for 30 min. Medium and floating cells were discarded, and adhered trophozoites were irradiated with 254 nm UV-C light at 150 J/m^2 ^for 8 s using a UV Stratalinker 1800 device (Stratagene). After treatment, cells were incubated in fresh TYI-S-33 medium at 37°C for 0.5, 3, 6 and 12 h to be used in different experiments. Non-irradiated cells were used as a control in all experiments. Cell viability was monitored by microscopy using a trypan blue dye exclusion test. Assays were done three times by duplicate.

### Evaluation of DNA fragmentation by TUNEL assay

Trophozoites (2 × 10^6^) were harvested at 0.5, 3, 6 and 12 h after UV-C irradiation, washed with PBS 1× and fixed with 1% paraformaldehyde. After cell permeabilization with 70% ethanol, DNA damage was quantified using the APO-BrdUTP TUNEL Assay Kit (Molecular Probes) in order to detect 3'-hydroxyl ends in DNA. Permeabilized trophozoites were incubated at 37°C for 1 h in the DNA-labeling solution, which contains terminal deoxynucleotidyl transferase enzyme (TdT) and deoxythymidine analog 5-bromo-2'-deoxyuridine 5'-triphosphate (BrdUTP). Then, cells were washed twice and suspended in antibody staining solution (Alexa Fluor 488 dye-labeled anti-BrdU antibody) at room temperature for 1 h. After that, cells were incubated in propidium iodide/RNase A staining buffer at room temperature for 30 min. Samples were analyzed by flow cytometry in a BD FACS Calibur system and fluorescence data were plotted with the FloJo software.

### Evaluation of DNA fragmentation by Comet assay

Trophozoites (5 × 10^4^) were harvested at 0.5, 3, 6 and 12 h after UV-C irradiation. Neutral comet assay were performed using protocols from Tice and co-workers [37]. Briefly, cells were mixed with agarose and spread over a warmed, precoated microscope slides. Agarose was allowed to solidify at 4°C, followed by immersion in cold lysis fresh solution (2.5 M NaCl, 100 mM EDTA, 10 mM Tris, pH 7) overnight. Next, electrophoresis was carried out in neutral buffer for 20 min at 1.5 V/cm (measured electrode to electrode) in the dark at 4°C. Finally, the slides were completely dried and ethidium bromide-stained DNA was observed at 400× magnification using an epifluorescence microscope (Leica DMIL).

### Detection of phosphorylated EhH2AX histones

The two *Ehh2ax *genes, which are homologous to the human *h2ax *gen, had been previously reported [22]. Their existence in the *E. histolytica *Pathema database [38] were confirmed by BLAST using yeast H2A and human H2AX protein sequences as queries. The presence of phosphorylated forms of EhH2AX histone (γEhH2AX) in *E. histolytica *protein extracts obtained 10 or 30 min after UV-C irradiation was evaluated by Western blot assays using the anti-phospho-Histone H2AX (pSer^139^), which was developed in rabbit using a synthetic phosphorylated peptide corresponding to 134–142 aa residues (including the phosphorylated Ser) of human H2AX histone C-terminus (Sigma). Subcellular fractionation to obtain CE and NE from clone A trophozoites was performed as described [39]. Proteins were separated by 10% SDS-PAGE, transferred to nitrocellulose membranes (BioRad) and blocked with 1% BSA/PBS solution. Then, filters were incubated at room temperature for 2 h with the anti-human γH2AX polyclonal antibody (1:7000 dilution), washed with PBS 1× 0.05%Tween and incubated at 37°C for 1 h with goat anti-rabbit IgG horseradish peroxidase secondary antibody (Zymed) at 1:10000 dilution. Bands were revealed by ECL Plus Western blotting system (Amersham). As internal controls, we used polyclonal antibodies (1:1000 dilution) raised against the *E. histolytica *poly(A) polymerase EhPAP and anti-actin antibodies.

### *In silico *identification of *E. histolytica *genes homologous to yeast RAD52 epistasis group

RAD52 epistasis group related genes were identified in *E. histolytica *Pathema database using both yeast and human protein sequences as queries. Putative *E. histolytica *orthologous proteins were selected from BLAST analysis according to the following criteria: (i) at least 20% identity and 35% homology to the query sequence; (ii) *e*-value lower than 0.002; and (iii) absence of stop codons in the coding sequence. Predicted aa sequences were aligned by the ClustalW software [40]. Functional domains were predicted by the Prosite program [41]. Phylogenetic inference was performed using the Neighbor-joining distance method [42] as implemented in the Molecular Evolutionary Genetics Analysis (MEGA version 3.1) software [43]. Tree robustness was established by bootstrapping test, involving 1000 replications of the data based on the criteria of 50% majority-rule consensus.

### RT-PCR assays

Total RNA was obtained using Trizol reagent (Invitrogen) from trophozoites of clone A grown in basal culture conditions or after UV-C treatment. Semi-quantitative RT-PCR was performed as previously described [44] using 1 μg of total RNA and specific primers for each gene (Table 2). As a control, we amplified a *25S rRNA *gene internal sequence. Products were separated by 6% PAGE, stained with ethidium bromide and submitted to densitometric analysis in a Gel doc 1000 apparatus (BioRad) using the Quantity One software. Data are the mean of three independent assays.

**Table 2:** Primers used in RT-PCR assays

Gene	Sense primer	Antisense primer	Amplified product (bp)	Tm (°C)
*Ehmre11*	5'-CGAGAAGAAGAGCAGCTCAA	5'-CTTTCCTTTTTCTTCAGCCA	391	49.5
*Ehrad50*	5'-CAGCCCAAGACATTCAAACA	5'-CTGCATAATTGTTGTGCCAA	277	49.5
*Ehnbs1*	5'-CACCTCCCACACCACAGTAT	5'-CTCCACCAATGAATGACCAT	549	49.0
*Ehrad51*	5'-ATTGCTTTTACACCAAAG	5'-TTCTTCTGAATTTAATCC	400	49.5
*Ehrad51C*	5'-CCACATGACATTGTGAGTCT	5'-GAATTATCCGATGAAGTGCT	155	45.0
*Ehrad52*	5'-ATGACTGAAATAGATACCTC	5'-AATTTGATTGTTTTAAGAAT	730	37.5
*Ehrad54*	5'-GTCATGCCATTGACCAATTA	5'-TCACACTCTTCCTCAGTTGG	499	47.5
*Ehrad54b*	5'-GGGCAAAAAATTCACCTAAA	5'-GTCGTGATCCTCCAAGTGCT	876	50.0
*Ehrad52/22*	5'-ATGTCTCATGAAATAAAACCAC	5'-TCATTTCTTACGTCTAACTATTACT	516	44.5
*25S rRNA*	5'-TATCAAATCAAAGGACCCGCT	5'-AAAAGA AAAACTAAGCGGTAA	631	51.0
*actin*	5'-AGCTGTTCTTTCATTATATGC	5'-TTCTCTTTCAGCAGTAGTGGT	220	48.0

### Cloning of the Ehrad51 gene

The 1098-bp full-length *Ehrad51 *gene was PCR-amplified from genomic DNA of clone A trophozoites using *Ehrad51-S *(5'-CGGGATCCAAAGTAATGAG TGCCAA GCA-3') sense and *Ehrad51-AS *(5'-CCAAGCTTGCCATTCTCC GTATTATGGC-3') antisense primers, which contain *Bam*HI and *Hind*III restriction sites, respectively (underlined). Amplification was performed as follows: 94°C for 5 min and 30 cycles at 94°*Cfor*35*s*,48°C for 35 s and 72°C for 1 min, plus a final extension step at 72°C for 7 min, using High Fidelity DNA *Taq *polymerase (Invitrogen). The PCR product was purified and cloned in frame into the pRSET A expression vector (Invitrogen). The recombinant pRSET *-Ehrad51 *plasmid construct was confirmed by automated DNA sequencing in an ABI-PRISM 310 (Applied Biosystem) sequencer.

### Expression and purification of recombinant EhRAD51 (rEhRAD51) protein

*E. coli *BL21 (DE3) pLysS bacteria were transformed with pRSET *-Ehrad51 *plasmid and grown at 37°C in 2-TY medium containing 100 μg/ml ampicillin and 34 μg/ml chloramphenicol to an OD_600nm _of 0.6. The expression of rEhRAD51 was induced with 1 mM isopropyl beta-D-thiogalacto pyranoside (IPTG) at 37°C for 3 h. Cells were harvested, resuspended in lysis buffer (50 mM NaH_2_PO_4_, 300 mM NaCl, 10 mM imidazole, pH 8.0) and lysed by sonication at 4°C. Soluble rEhRAD51 was purified near to homogeneity under denaturing and native conditions through Ni^2+^-NTA affinity chromatography according to the manufacturer recommendations (Qiagen). Purified rEhRAD51 identity and integrity were confirmed by 10% SDS-PAGE and Western blot assays using anti-6xHis tag antibodies (Roche) at 1:5000 dilution and the ECL Plus Western blotting detection system (Amersham).

### Production of polyclonal antibodies raised against EhRAD51

Purified rEhRAD51 was submitted to preparative 10% SDS-PAGE, electroeluted from Coomassie stained-gels and subsequently used as antigen to immunize a New Zeland male rabbit. An initial dose of 200 μg of rEhRAD51 in complete Freund's adjuvant (Sigma) was subcutaneously inoculated into the animal, and then three doses of 100 μg in incomplete Freund's adjuvant were injected every 15 days. One week after the last immunization, the rabbit was bled and polyclonal antiserum was obtained. IgGs were purified through protein G sepharose chromatography and tested for reactivity against rEhRAD51 protein by Western blot assays.

### Immunodetection of EhRAD51 in subcellular extracts

Western blot assays were performed using CE and NE proteins obtained before or 30 min after UV-C irradiation, and the membranes were incubated with anti-EhRAD51 polyclonal antibodies (1:1000 dilution) and goat anti-rabbit IgG horseradish peroxidase secondary antibody (Zymed)(1:10000 dilution). Immunodetected proteins were revealed with the ECL Plus Western blotting system (Amersham). The specificity of the anti-EhRAD51 antibodies was confirmed using anti-EhRAD51 antibodies pre-incubated with purified rEhRAD51 protein. As internal controls, we used polyclonal antibodies raised against the *E. histolytica *EhPAP [23] and actin proteins.

### Laser confocal microscopy assays

Trophozoites were grown on sterile coverslips, fixed with 4% paraformaldehyde at 37°C for 1 h, permeabilized with acetone and blocked with 1% BSA/PBS. Next, cells were incubated with anti-EhRAD51 polyclonal antibodies (1:200 dilution) at 37°C for 2 h, followed by the anti-rabbit fluoresceinated monoclonal antibody (1:100 dilution) at 37°C for 1 h. Then, trophozoites were washed three times with PBS 1× at room temperature and DNA was counterstained with propidium iodide (5 μg/ml) for 7 min. Light optical sections were obtained through a Nikon inverted microscope attached to a laser confocal scanning system (Leica) and analyzed by Confocal Assistant software Image J [45].

### DNA-binding assays

For the electrophoretic mobility shift assay (EMSA), we used two DNA probes: a 50-nt ss oligonucleotide (*adh*50) from the *Ehadh112 *gene [46], which was [γ-^32^P]dATP (500 μCi/mmol) 3'-end labeled by T4 polynucleotide kinase at 37°C for 30 min, and a 270-bp dsDNA fragment (*pgp*270) of the 3'-UTR *EhPgp5 *gene [50] that was [α-^32^P]dATP (200 μCi/mmol) uniformly labeled by PCR. EMSA was carried out in a 25 μl final volume reaction in binding buffer (50 mM Tris-HCl pH 7.8, 1 mM DTT, 10 mM MgCl_2_, 1 mM ATP) in the presence of increasing amounts of native rEhRAD51 (0, 2.5, 5 and 7.5 μg). Reactions were started by addition of *adh*50 or *pgp*270 radiolabeled probes (10000 cpm) at 37°C for 15 min. Control assays were performed substituting purified rEhRAD51 by the mock purified fraction obtained from untransformed bacteria. DNA-protein complexes were resolved on 6% non-denaturing TBE polyacrylamide gels, vacuum-dried and exposed to Phosphor Imager screen (BioRad).

### D-loop structure formation assay

The EhRAD51 homologous DNA strand transfer activity was evaluated by the D-loop formation assay according to the described procedure [47]. A ssDNA fragment of 200 bases (*pgp*200), which is complementary to the 3'-UTR *Ehpgp5 *gene cloned in the dsDNA plasmid [44], was [γ-^32^P]dATP (500 μCi/mmol) 3'-end labeled by T4 polynucleotide kinase at 37°C for 30 min. Increasing amounts of rEhRAD51 (0, 2.5, 5 and 7.5 μg) were pre-incubated in reaction buffer (50 mM Tris-HCl pH 7.8, 1 mM DTT, 10 mM MgCl_2 _and 1 mM ATP) with the *pgp*200 probe (10,000 cpm) at 37°C for 15 min. Then, homologous dsDNA plasmid (1 μM) was added and the mixture was incubated at 37°C for 30 min. A non-related plasmid was used as heterologous dsDNA control. Reactions were stopped by addition of 0.1% SDS. To prevent that EhRAD51 binds and shifts the *pgp*200 probe, samples were deproteinized with proteinase K (1 mg/ml) at 37°C for 10 min. Then, they were fractionated by 1% agarose gel electrophoresis in TAE 1× buffer and transferred to a nylon membrane (Amersham) in SSC 20× solution overnight. Homologous DNA strand transfer activity of rEhRAD51 was evaluated through the visualization of radioactive DNA products in a Phosphor Imager (BioRad) and quantified by densitometry using the Quantity one software (BioRad).

## Authors' contributions

MLC carried out most of the experiments and drafted the manuscript. EO participated in the study design, data interpretation and co-wrote the manuscript. Most of experiments presented here were performed in EO laboratory (CINVESTAV-IPN). LAM participated in the study design, data interpretation and bioinformatic analysis. CLC conceived the project, cloned the *Ehrad51 *gene, supervised the experiments and co-wrote the manuscript. All authors read and approved the final manuscript.

## Supplementary Material

Additional file 1 Comparisons of EhRAD51 with orthologous proteins from other organisms. This table includes proteins homologous to EhRAD51 with respective homology/identity and e-values. Click here for file

Additional file 2 Predictions of EhRAD51 tertiary structure using the Swiss Model software and the yeast RAD51 protein crystal structure (PDB entry 1szp) as template. **A**. Predicted three-dimensional model of EhRAD51 protein showing the N-terminal domain (ND) constituted by a five-α helix bundle (α1 to α5) and an ATPase domain (AD) conformed by a twisted central β-sheet, which includes 10 β strands sandwiched by α-helices on both sides connected by a polymerization motif (PM). **B**. ATPase Walker A motif lies between β1 and α8 and conserves the catalytic lysine (K160) and threonine (T160) residues, which are associated to ATP γ-phosphate contact and Mg^2+ ^ion stabilization, respectively, in homologous proteins. Walker B motif lies on β4 and precedes α12 and the disordered DNA-binding loop 1. ATP cap is in close proximity to an ATP molecule. EhRAD51 DNA-binding loop 2 is formed by two inter-connected β strands (β6 and β7). **C**. Three-dimensional representation of Polymerization motif (PM). Critical conserved residues conforming PM in helix 6 are shown. Key motifs were colored as follow: violet, PM; red, ATPase Walker A; green, Walker B; blue, ATP cap; yellow, Loop 1 and purple, Loop 2. Models were displayed and refined using the Pymol PBD viewer. Click here for file

## References

[B1] Jackson T, Reddy S, Fincham J, bd-Alla M, Welles S, Ravdin J (2000). A comparison of cross-sectional and longitudinal seroepidemiological assessments of entamoeba-infected populations in South Africa. Arch Med Res.

[B2] Orozco E, de la Cruz HF, Rodriguez MA (1985). Isolation and characterization of Entamoeba histolytica mutants resistant to emetine. Mol Biochem Parasitol.

[B3] Zaki M, Meelu P, Sun W, Clark CG (2002). Simultaneous differentiation and typing of Entamoeba histolytica and Entamoeba dispar. J Clin Microbiol.

[B4] Báez-Camargo M, Gharaibeh R, Riverón AM, de la Cruz Hernández F, Luna JP, Gariglio P, Chávez P, Orozco E (1996). Gene amplification in Entamoeba histolytica. Invasion Metastasis.

[B5] Lisby M, Rothstein R (2004). DNA damage checkpoint and repair centers. Curr Opin Cell Biol.

[B6] Wei C, Skopp R, Takata M, Takeda S, Price CM (2002). Effects of double-strand break repair proteins on vertebrate telomere structure. Nucleic Acids Res.

[B7] Masson JY, West SC (2001). The Rad51 and Dmc1 recombinases: a non-identical twin relationship. Trends Biochem Sci.

[B8] van Attikum H, Gasser SM (2005). The histone code at DNA breaks: a guide to repair?. Nat Rev Mol Cell Biol.

[B9] Longhese MP, Mantiero D, Clerici M (2006). The cellular response to chromosome breakage. Mol Microbiol.

[B10] Peck RF, DasSarma S, Krebs MP (2000). Homologous gene knockout in the archaeon Halobacterium salinarum with ura3 as a counterselectable marker. Mol Microbiol.

[B11] Symington LS (2002). Role of RAD52 epistasis group genes in homologous recombination and double-strand break repair. Microbiol Mol Biol Rev.

[B12] Thacker J (2005). The RAD51 gene family, genetic instability and cancer. Cancer Lett.

[B13] Paques F, Haber JE (1999). Multiple pathways of recombination induced by double-strand breaks in Saccharomyces cerevisiae. Microbiol Mol Biol Rev.

[B14] Tashiro S, Walter J, Shinohara A, Kamada N, Cremer T (2000). Rad51 accumulation at sites of DNA damage and in postreplicative chromatin. J Cell Biol.

[B15] Bhattacharyya MK, Norris DE, Kumar N (2004). Molecular players of homologous recombination in protozoan parasites: implications for generating antigenic variation. Infect Genet Evol.

[B16] Conway C, Proudfoot C, Burton P, Barry JD, McCulloch R (2002). Two pathways of homologous recombination in Trypanosoma brucei. Mol Microbiol.

[B17] Dzikowski R, Deitsch K (2006). Antigenic variation by protozoan parasites: insights from Babesia bovis. Mol Microbiol.

[B18] Freitas-Junior LH, Bottius E, Pirrit LA, Deitsch KW, Scheidig C, Guinet F, Nehrbass U, Wellems TE, Scherf A (2000). Frequent ectopic recombination of virulence factor genes in telomeric chromosome clusters of P. falciparum. Nature.

[B19] Triglia T, Foote SJ, Kemp DJ, Cowman AF (1991). Amplification of the multidrug resistance gene pfmdr1 in Plasmodium falciparum has arisen as multiple independent events. Mol Cell Biol.

[B20] Woods WG, Dyall-Smith ML (1997). Construction and analysis of a recombination-deficient (radA) mutant of Haloferax volcanii. Mol Microbiol.

[B21] Chen HT, Bhandoola A, Difilippantonio MJ, Zhu J, Brown MJ, Tai X, Rogakou EP, Brotz TM, Bonner WM, Ried T, Nussenzweig A (2000). Response to RAG-mediated VDJ cleavage by NBS1 and gamma-H2AX. Science.

[B22] Sullivan WJ, Naguleswaran A, Angel SO (2006). Histones and histone modifications in protozoan parasites. Cell Microbiol.

[B23] Garcia-Vivas J, Lopez-Camarillo C, Azuara-Liceaga E, Orozco E, Marchat LA (2005). Entamoeba histolytica: cloning and expression of the poly(A) polymerase EhPAP. Exp Parasitol.

[B24] Bell CE (2005). Structure and mechanism of Escherichia coli RecA ATPase. Mol Microbiol.

[B25] Wu Y, Qian X, He Y, Moya IA, Luo Y (2005). Crystal structure of an ATPase-active form of Rad51 homolog from Methanococcus voltae. Insights into potassium dependence. J Biol Chem.

[B26] Shin DS, Pellegrini L, Daniels DS, Yelent B, Craig L, Bates D, Yu DS, Shivji MK, Hitomi C, Arvai AS, Volkmann N, Tsuruta H, Blundell TL, Venkitaraman AR, Tainer JA (2003). Full-length archaeal Rad51 structure and mutants: mechanisms for RAD51 assembly and control by BRCA2. EMBO J.

[B27] Conway AB, Lynch TW, Zhang Y, Fortin GS, Fung CW, Symington LS, Rice PA (2004). Crystal structure of a Rad51 filament. Nat Struct Mol Biol.

[B28] Birrell GW, Brown JA, Wu HI, Giaever G, Chu AM, Davis RW, Brown JM (2002). Transcriptional response of Saccharomyces cerevisiae to DNA-damaging agents does not identify the genes that protect against these agents. Proc NatlAcad Sci USA.

[B29] Garinis GA, Mitchell JR, Moorhouse MJ, Hanada K, de Waard H, Vandeputte D, Jans J, Brand K, Smid M, van der Spek PJ, Hoeijmakers JH, Kanaar R, van der Horst GT (2005). Transcriptome analysis reveals cyclobutane pyrimidine dimers as a major source of UV-induced DNA breaks. EMBO J.

[B30] Campbell C, Romero DP (1998). Identification and characterization of the RAD51 gene from the ciliate Tetrahymena thermophila. Nucleic Acids Res.

[B31] McCready S, Muller JA, Boubriak I, Berquist BR, Ng WL, Dassarma S (2005). UV irradiation induces homologous recombination genes in the model archaeon, Halobacterium sp. NRC-1. Saline Systems.

[B32] Sørensen CS, Hansen LT, Dziegielewski J, Syljuåsen RG, Lundin C, Bartek J, Helleday T (2005). The cell-cycle checkpoint kinase Chk1 is required for mammalian homologous recombination repair. Nat Cell Biol.

[B33] Haaf T, Golub EI, Reddy G, Radding CM, Ward DC (1995). Nuclear foci of mammalian Rad51 recombination protein in somatic cells after DNA damage and its localization in synaptonemal complexes. Proc Natl Acad Sci USA.

[B34] Kant CR, Rao BJ, Sainis JK (2005). DNA binding and pairing activity of OsDmc1, a recombinase from rice. Plant Mol Biol.

[B35] Tombline G, Heinen CD, Shim KS, Fishel R (2002). Biochemical characterization of the human RAD51 protein. III. Modulation of DNA binding by adenosine nucleotides. J Biol Chem.

[B36] Orozco E, Suarez ME, Sanchez T (1985). Differences in adhesion, phagocytosis and virulence of clones from Entamoeba histolytica, strain HM1:IMSS. Int J Parasitol.

[B37] Tice RR, Agurell E, Anderson D, Burlinson B, Hartmann A, Kobayashi H, Miyamae Y, Rojas E, Ryu JC, Sasaki YF (2000). Single cell gel/comet assay: guidelines for in vitro and in vivo genetic toxicology testing. Environ Mol Mutagen.

[B38] The *E. histolytica *Pathema datatabase. http://pathema.tigr.org/tigr-scripts/Entamoeba/PathemaHomePage.cgi.

[B39] Marchat LA, Pezet-Valdez M, Lopez-Camarillo C, Orozco E (2003). Entamoeba histolytica: expression and DNA binding of CCAAT/enhancer-binding proteins are regulated through the cell cycle. Exp Parasitol.

[B40] ClustalW software. http://www.ebi.ac.uk/clustalw/.

[B41] Prosite program. http://www.expasy.org/tools/scanprosite/.

[B42] Saitou N, Nei M (1987). The neighbor-joining method: a new method for reconstructing phylogenetic trees. Mol Biol Evol.

[B43] MEGA version 3.1 software. http://www.megasoftware.net.

[B44] Lopez-Camarillo C, Luna-Arias JP, Marchat LA, Orozco E (2003). EhPgp5 mRNA stability is a regulatory event in the Entamoeba histolytica multidrug resistance phenotype. J Biol Chem.

[B45] Confocal Assistant software Image J. http://rsb.info.nih.gov/ij/.

[B46] Banuelos C, Garcia-Rivera G, Lopez-Reyes I, Orozco E (2005). Functional characterization of EhADH112: an Entamoeba histolytica Bro1 domain-containing protein. Exp Parasitol.

[B47] Kinebuchi T, Kagawa W, Enomoto R, Tanaka K, Miyagawa K, Shibata T, Kurumizaka H, Yokoyama S (2004). Structural basis for octameric ring formation and DNA interaction of the human homologous-pairing protein Dmc1. Mol Cell.

