# Supplementary material for: Transcriptional profile of the homologous recombination machinery and characterization of the EhRAD51 recombinase in response to DNA damage in Entamoeba histolytica
Source: BMC Mol Biol. 2008 Apr 10;9:35. doi: 10.1186/1471-2199-9-35 (PMC2324109; doi:10.1186/1471-2199-9-35)
Supplement: Additional file 1 — Comparisons of EhRAD51 with orthologous proteins from other organisms. This table includes proteins homologous to EhRAD51 with respective homology/identity and e-values. [file 1471-2199-9-35-S1.doc]

**Additional file 1.** Comparisons of EhRAD51 with orthologous proteins from other organisms.

^a^ Swiss-Prot/TrEMBL databases

| Organism | Protein | Accession  number ^a^ | Size  (aa) | I  (%) | H  (%) | e-value |
| --- | --- | --- | --- | --- | --- | --- |
| *Homo sapiens* | RAD51 | Q06609 | 339 | 75 | 85 | 3e-29 |
| *Mus musculus* | RAD51 | Q08297 | 339 | 75 | 85 | 3e-29 |
| *Trypanosoma brucei* | RAD51 | Q9U6W1 | 373 | 68 | 78 | 3e-23 |
| *Leishmania major* | RAD51 | O61127 | 377 | 65 | 78 | 3e-22 |
| *Arabidopsis thaliana* | RAD51 | P94102 | 342 | 62 | 78 | 7e-22 |
| *Saccharomyces cerevisae* | RAD51 | P25454 | 400 | 59 | 77 | 2e-20 |
| *Methanococcus voltae* | RADA | O73948 | 322 | 51 | 74 | 9e-16 |
| *Escherichia coli* | RECA | P0A7G9 | 352 | 36 | 45 | 0.0075 |

I, identity; H, homology
