# Supplementary figures and images for: Transcriptional profile of the homologous recombination machinery and characterization of the EhRAD51 recombinase in response to DNA damage in Entamoeba histolytica
Source: BMC Mol Biol. 2008 Apr 10;9:35. doi: 10.1186/1471-2199-9-35 (PMC2324109; doi:10.1186/1471-2199-9-35)

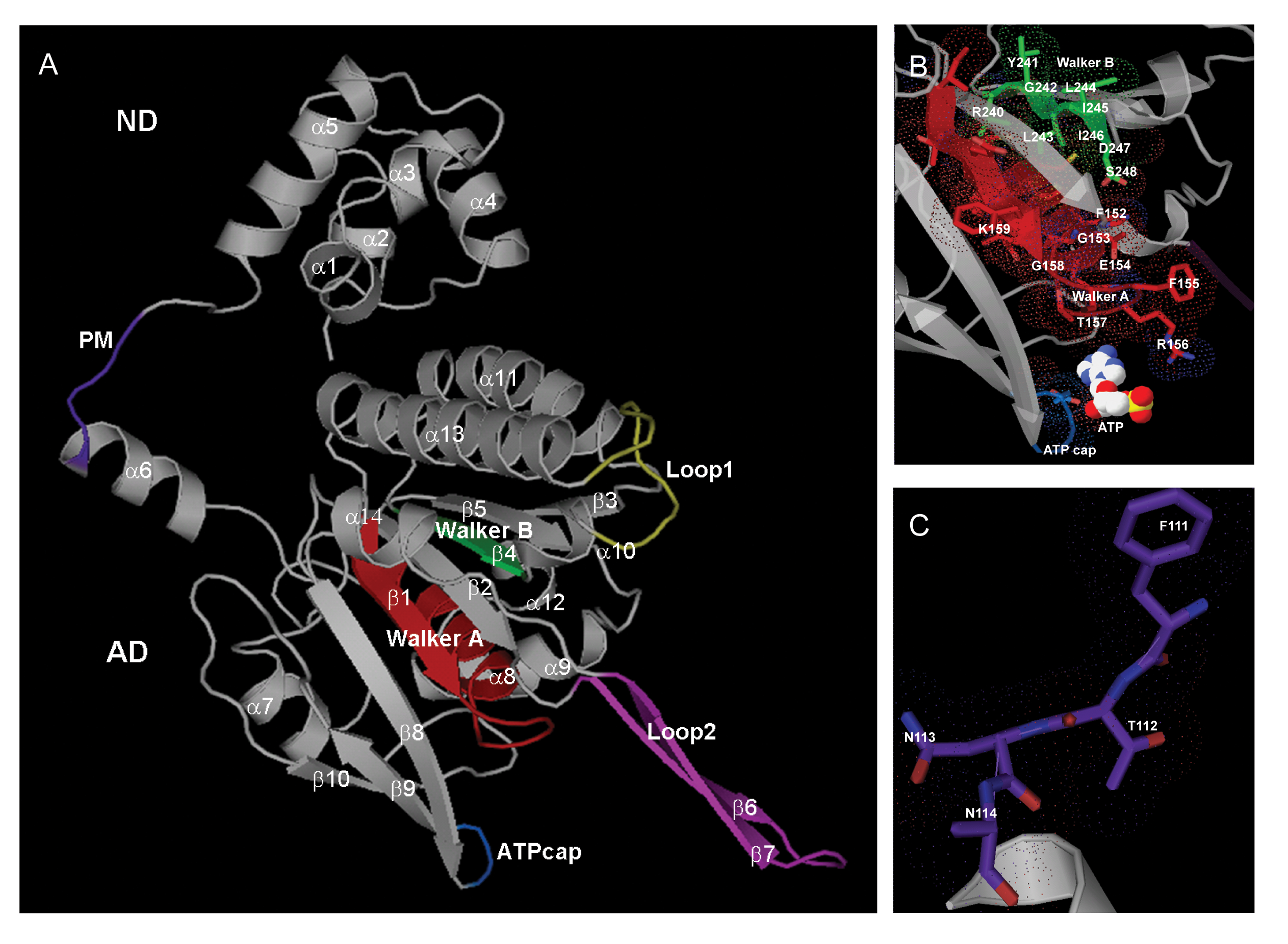

Supplement: Additional file 2 — Predictions of EhRAD51 tertiary structure using the Swiss Model software and the yeast RAD51 protein crystal structure (PDB entry 1szp) as template. A. Predicted three-dimensional model of EhRAD51 protein showing the N-terminal domain (ND) constituted by a five-α helix bundle (α1 to α5) and an ATPase domain (AD) conformed by a twisted central β-sheet, which includes 10 β strands sandwiched by α-helices on both sides connected by a polymerization motif (PM). B. ATPase Walker A motif lies between β1 and α8 and conserves the catalytic lysine (K160) and threonine (T160) residues, which are associated to ATP γ-phosphate contact and Mg2+ ion stabilization, respectively, in homologous proteins. Walker B motif lies on β4 and precedes α12 and the disordered DNA-binding loop 1. ATP cap is in close proximity to an ATP molecule. EhRAD51 DNA-binding loop 2 is formed by two inter-connected β strands (β6 and β7). C. Three-dimensional representation of Polymerization motif (PM). Critical conserved residues conforming PM in helix 6 are shown. Key motifs were colored as follow: violet, PM; red, ATPase Walker A; green, Walker B; blue, ATP cap; yellow, Loop 1 and purple, Loop 2. Models were displayed and refined using the Pymol PBD viewer. [file 1471-2199-9-35-S2.tiff]
